# The evolutionary maintenance of ancient recombining sex chromosomes in the ostrich

**DOI:** 10.1371/journal.pgen.1010801

**Published:** 2023-06-30

**Authors:** Homa Papoli Yazdi, Colin Olito, Takeshi Kawakami, Per Unneberg, Mads F. Schou, Schalk W. P. Cloete, Bengt Hansson, Charlie K. Cornwallis

**Affiliations:** 1 Department of Biology, Lund University, Lund, Sweden; 2 Science for Life Laboratory, Department of Cell and Molecular Biology, Uppsala University, Uppsala, Sweden; 3 Embark Veterinary, Inc., Boston, Massachusetts, United States of America; 4 Department of Cell and Molecular Biology, National Bioinformatics Infrastructure Sweden, Science for Life Laboratory, Uppsala University, Uppsala, Sweden; 5 Directorate Animal Sciences, Western Cape Department of Agriculture, Elsenburg, South Africa; 6 Department of Animal Sciences, Stellenbosch University, Matieland, South Africa; Geisel School of Medicine at Dartmouth, UNITED STATES

## Abstract

Sex chromosomes have evolved repeatedly across the tree of life and often exhibit extreme size dimorphism due to genetic degeneration of the sex-limited chromosome (e.g. the W chromosome of some birds and Y chromosome of mammals). However, in some lineages, ancient sex-limited chromosomes have escaped degeneration. Here, we study the evolutionary maintenance of sex chromosomes in the ostrich (*Struthio camelus*), where the W remains 65% the size of the Z chromosome, despite being more than 100 million years old. Using genome-wide resequencing data, we show that the population scaled recombination rate of the pseudoautosomal region (PAR) is higher than similar sized autosomes and is correlated with pedigree-based recombination rate in the heterogametic females, but not homogametic males. Genetic variation within the sex-linked region (SLR) (π = 0.001) was significantly lower than in the PAR, consistent with recombination cessation. Conversely, genetic variation across the PAR (π = 0.0016) was similar to that of autosomes and dependent on local recombination rates, GC content and to a lesser extent, gene density. In particular, the region close to the SLR was as genetically diverse as autosomes, likely due to high recombination rates around the PAR boundary restricting genetic linkage with the SLR to only ~50Kb. The potential for alleles with antagonistic fitness effects in males and females to drive chromosome degeneration is therefore limited. While some regions of the PAR had divergent male-female allele frequencies, suggestive of sexually antagonistic alleles, coalescent simulations showed this was broadly consistent with neutral genetic processes. Our results indicate that the degeneration of the large and ancient sex chromosomes of the ostrich may have been slowed by high recombination in the female PAR, reducing the scope for the accumulation of sexually antagonistic variation to generate selection for recombination cessation.

## Author summary

In birds, sex is determined by a pair of sex chromosomes where females are ZW and males are ZZ. In many taxa, the W chromosome has degenerated except for a small part that continues to recombine in both sexes called the pseudoautosomal region (PAR). The prominent theory for why recombination suppression between Z and W evolves is that selection favours linkage between the sex-determination locus and alleles with sex-specific fitness effects. Recombination cessation results in reduced selection efficiency and ultimately the degeneration of the W chromosome. There are, however, some ancient sex chromosomes that deviate from this path of W demise that present one of the fascinating puzzles of sex chromosome evolution. In ostriches, sex chromosomes with a long PAR have been maintained for over 100 million years. Here, we show that high recombination rates in the region immediately adjacent to the PAR boundary break the association between the sex-linked region and PAR loci. This suggests that the integrity of sex chromosomes may be maintained in species where high recombination rates minimize the influence of sex linkage on neighboring genes, reducing the likelihood that mutations with sex-specific fitness effects accumulate.

## Introduction

**Fig 1:**
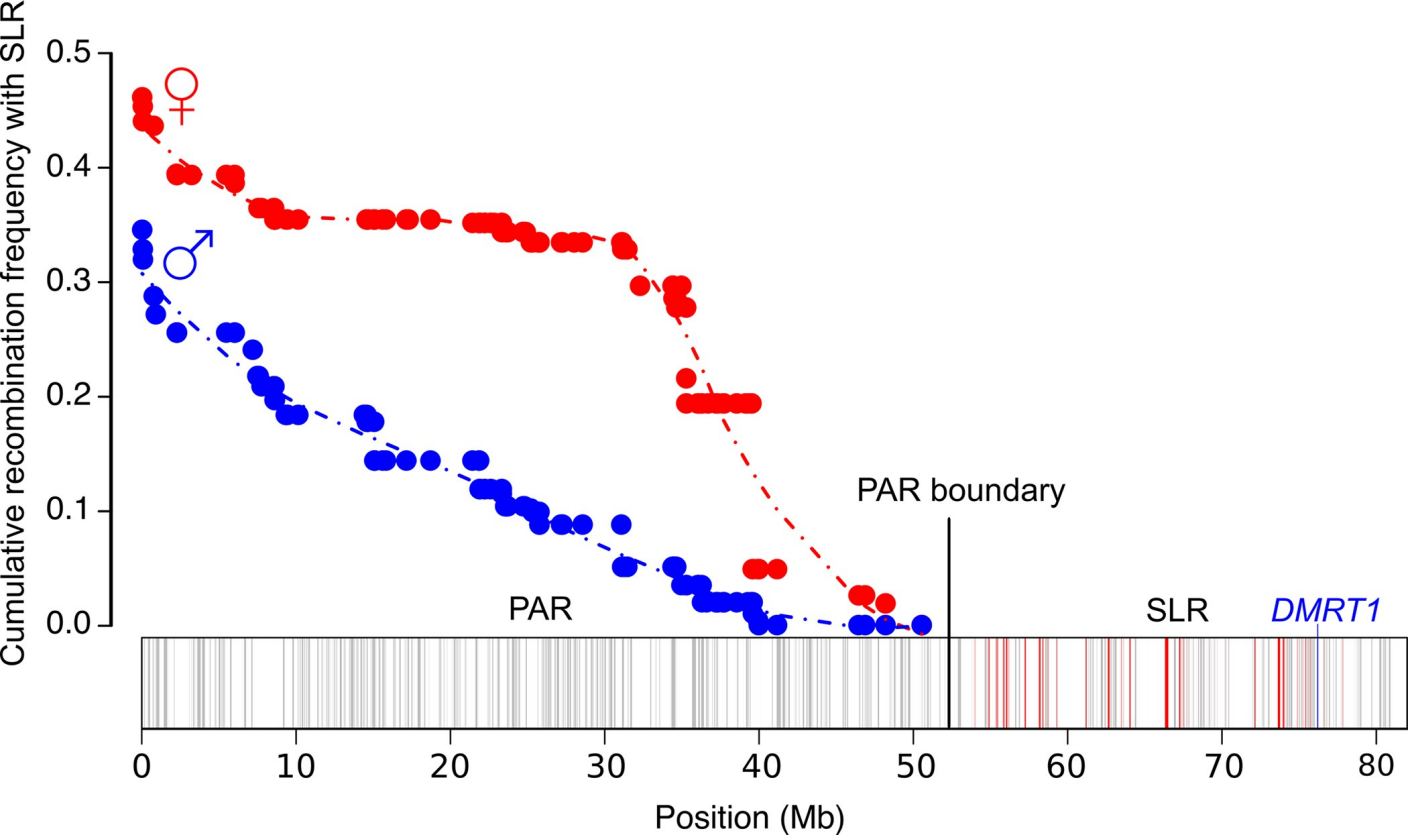
The structure of the ostrich Z chromosome. The Z chromosome consists of the PAR and the SLR. In the PAR, sex chromosomes recombine in both males (Z/Z) and females (Z/W), while in the SLR recombination only occurs in males. The cumulative recombination frequency between the PAR boundary (at ~52.2 Mb) and loci within the PAR determines the extent of sex-linked inheritance, and is calculated using the female and male genetic map lengths (80.628 cM and 42.641 cM, respectively from [10]) using the Kosambi map function [11]. Gray bars represent genes along the Z chromosome. Red bars indicate homologous genes that are still present within the SLR on the W chromosome (i.e., gametologous genes). *DMRT1* is the Z-linked avian sex-determining gene [12] (blue bar).

In many taxa, sex is determined by genes residing on a pair of homologous chromosomes, such as the XY chromosomes of mammals and the ZW chromosomes of birds [1]. These sex chromosomes typically have two regions, a sex-linked region (SLR) where sex-determining genes are located and recombination is suppressed, and one or more pseudoautosomal regions (PAR) where recombination persists to ensure proper chromosome pairing during meiosis (Fig 1) [2,3]. Interestingly, the size of the PAR differs widely among even closely related species, demonstrating the dynamic evolution of recombination on sex chromosomes [3]. However, it remains unclear why some species have sex chromosomes with small PARs and extensive non-recombining regions [4–6], while others have large PARs that are maintained over long evolutionary periods [7–9].

A popular explanation for why recombination suppression spreads across sex chromosomes and PARs degrade, is based on sexually antagonistic selection [13]. Alleles that are beneficial when expressed in one sex, but deleterious when expressed in the other (i.e., sexually antagonistic variation) cause indirect selection for suppressed recombination between sex chromosomes. This results in female-beneficial alleles becoming associated with the W and male-beneficial alleles becoming associated with the Z [14]. Importantly, the conditions for maintaining such sexually antagonistic variation are more permissive when loci are partially genetically linked to the SLR [13]. At the same time, stronger sex-linkage increases the coalescence times of sampled alleles from the two sex chromosomes. This leads to increased neutral diversity at partially sex-linked sites within the PAR [15], and increases the potential for the accumulation of sexually antagonistic genetic variation [16].

A key process determining levels of genetic linkage between PAR loci and the SLR is the rate of recombination. High recombination rates close to the SLR are expected to reduce the proportion of PAR loci with sex-biased inheritance and the potential for sexually antagonistic variation to accumulate. Recombination rates can also impact other processes that influence the evolution of genetic variation on sex chromosomes [17]. For example, regions with low recombination, particularly when enriched in functional elements under selection, can show large reductions in neutral genetic diversity due to the impact of selection at linked sites [18,19]. In contrast, regions with high recombination rates will be impacted by GC-biased gene conversion which can also resemble the effect of selection [20–22]. Quantifying recombination rates across the PAR in relation to the SLR is therefore crucial to understand the potential for sexually antagonistic selection, patterns of neutral genetic diversity and the evolution of expanded non-recombining regions.

Molecular and population genetic studies of species with highly degenerated W/Y chromosomes have revealed several characteristics of small PARs. In particular, the heterogametic sex (ZW females or XY males) typically has extreme recombination rates. In collared flycatchers (*Ficedula albicollis*), the PAR is only 630 Kb (1.05% of the Z length) and has a female recombination rate that is about 200 times higher than the genome-wide average, resulting in a GC-rich sequence due to GC-biased gene conversion [5]. Similarly, in humans, PAR1 is about 2.7 Mb (1.73% of the X length) with a male recombination rate that is ~17 times higher than the autosomal average [23]. The role of sexual antagonism in the expansion of the SLR in these systems has been investigated by estimating the divergence in allele frequencies between males and females and examining sex-biased gene expression, which can be a sign of resolved sexual conflict [24]. In the human PAR1, there are divergent allele frequencies between the sex chromosomes and enrichment of male-biased expressed genes, but these patterns are not entirely explained by sexual antagonism [25,26]. However, in collared flycatchers, there is little evidence of male-female allele frequency divergence and sex-biased gene expression. This is potentially due to a recombination hotspot close to the PAR-SLR boundary reducing the opportunity for sexually antagonistic genetic variation to accumulate [5]. In contrast to these examples of small PARs, the population genetic dynamics of ancient sex chromosomes with large PARs remain largely unknown.

Sex chromosomes with long PARs are found in both animals [9,27,28] and dioecious plants [29]. Some of these sex chromosomes, such as those found in the flowering plant *Silene latifolia* [30], have evolved relatively recently and are in the early stages of degeneration. Sex chromosomes with long and ancient PARs are relatively rare, but notable examples occur within the avian lineage Palaeognathae (tinamous and ratites). In Palaeognathae, the sex chromosomes first evolved more than 100 MYA in a shared common ancestor with the Neognathae, which contains >99% of extant avian species [31]. Unlike the Neognathae, many Palaeognathae species, particularly the ratites, have retained long PARs ranging from between ~65% to 73% of the length of the Z chromosome [9,32–35]. For example, the PAR of the ostrich is ~52.2 Mb which comprises ~65% of the Z chromosome. Molecular evolution studies of the ostrich PAR have shown that rates of evolution, measured by synonymous substitution rates, are similar to autosomal sequences and that the PAR is not enriched for male-biased expressed genes [9]. Recombination frequency along the PAR obtained from an ostrich pedigree also showed a higher frequency of recombination along the PAR in females compared to males (Fig 1) [10]. However, the resolution of the markers along the Z chromosome was low with an average of 1 marker every ~250Kb. It is therefore unclear how the fine-scale recombination landscape of the ostrich PAR influences levels of genetic variation and the degree of genetic linkage with the SLR, a critical factor determining the scope for sexually antagonistic alleles to accumulate.

Here we use whole-genome re-sequencing and a genetic linkage map [10] of the ostrich sex chromosomes to test if recombination rate can help explain the evolutionary maintenance of the large, ancient PAR. First, we investigate the pattern of recombination across the sex chromosomes and its influence on the degree of genetic linkage with the SLR (measured by linkage disequilibrium (LD)). We also compare sex-averaged population recombination rates to male and female estimates from the genetic linkage map to quantify how historical recombination rates have been shaped by each sex. Second, we test how recombination rates influence genetic diversity and GC content and if this relates to gene density across the PAR. Third, we examine if there are regions of the PAR that potentially harbor sexually antagonistic alleles by calculating allele frequency differences between males and females. Finally, we evaluate whether patterns of genetic diversity and the observed differences in allele frequencies between males and females across the PAR, particularly in the region close to SLR, are consistent with predictions from population genetic theory using coalescent simulations based on [15].

## Results

### Recombination rate and linkage disequilibrium across the sex chromosomes

**Fig 2:**
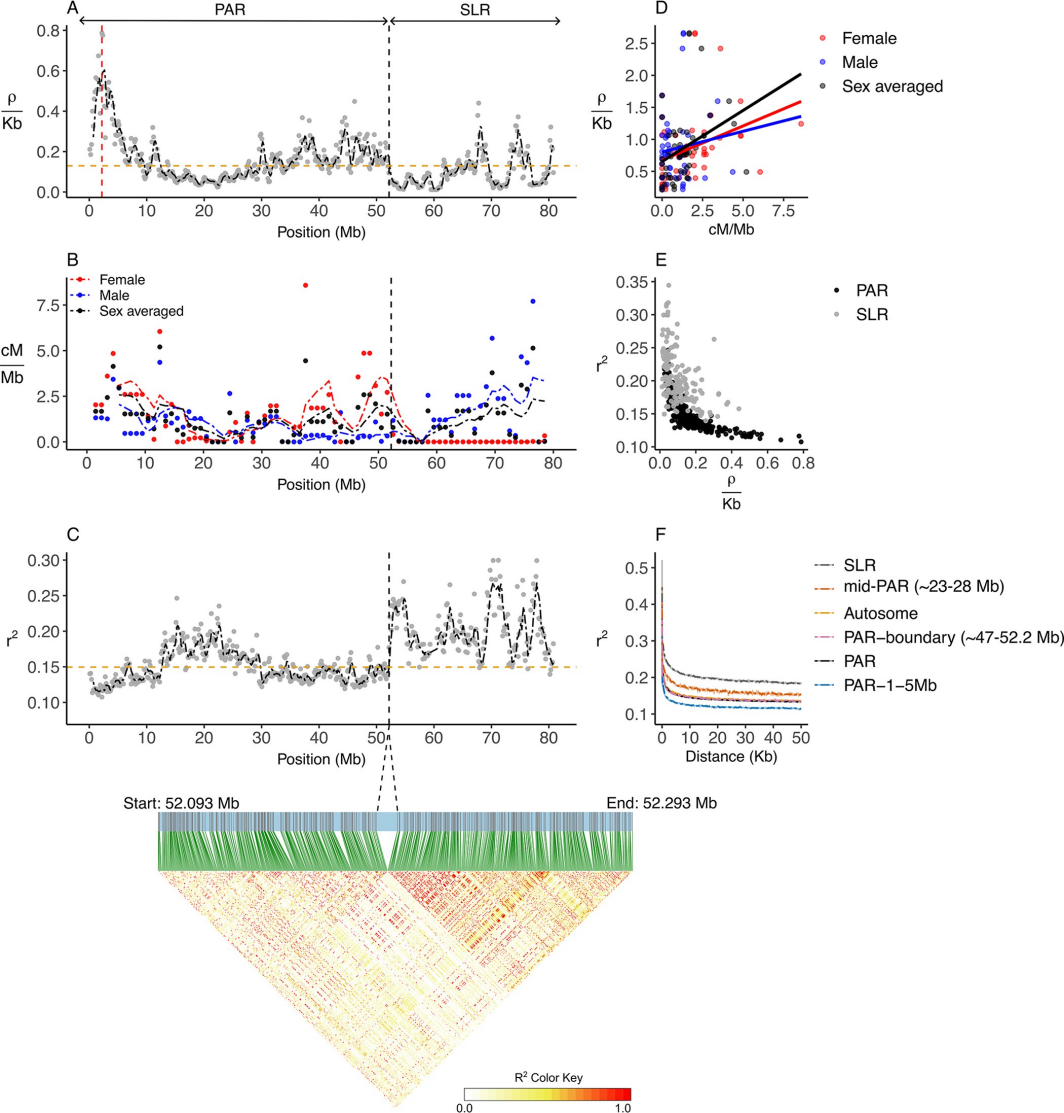
Recombination rate and linkage disequilibrium (LD) across the ostrich Z chromosome. (A) Population scaled recombination rate (*ρ* = 4*N*_*e*_*r*) calculated from SNPs in windows of 200 Kb with 50 Kb overlap using LDhat [36] (Gray points). The horizontal dashed line in orange is the autosomal average for *ρ*/*Kb* = 0.13. The vertical dashed line in red at 2.2 Mb represents the maximum *ρ*/*Kb* across the PAR. The black dash-dotted line is the rolling average calculated over a span of ~1 Mb (B) Female, male and sex-averaged recombination rate (cM/Mb) in windows of 1 Mb obtained from a genetic linkage map [10]. The dash-dotted lines are the rolling averages calculated over a span of ~5 Mb (C) LD in windows of 200 Kb with 50 Kb overlap (Gray points). The horizontal dashed line in orange indicates the average autosomal LD (*r*^2^ = 0.15). The black dash-dotted line is the rolling average calculated over a span of ~1 Mb. The triangular matrix plot indicates LD across the 200 Kb of the PAR boundary. The vertical dashed lines in A to C at ~52.2 Mb represents the PAR boundary. (D) Population scaled recombination rate (*ρ*/*Kb*) was significantly correlated to female (*R* = 0.35, *p* = 0.013) and sex-averaged (*R* = 0.32, *p* = 0.021) genetic map recombination rate (cM/Mb) across the PAR, but not to male recombination rate (*R* = 0.1, *p* = 0.45). Each dot represents an average for 1 Mb windows. (E) LD steeply declined with population scaled recombination rate (*ρ*/*Kb*) for the PAR and the SLR. (F) LD decay in relation to pairwise SNP distance across the SLR, mid-PAR (~23–28 Mb), autosomes, PAR-boundary (~47–52.2 Mb), the PAR and PAR-1-5 Mb.

The sex-averaged population recombination rate (*ρ* = 4*N*_*e*_*r)* across the PAR (mean (SD): *ρ*/Kb = 0.17 (0.12)) was significantly higher than on similar sized autosomes (mean (SD): *ρ*/Kb = 0.13 (0.071)) (Mann-Whitney U: U = 40087, *p* = 5.867e-05). This is expected given crossovers must occur in a smaller chromosomal segment of the female PAR. Recombination rates were highly variable across the sex chromosomes but could be divided into four regions with significantly different recombination rate regimes based on a change-point analysis (estimated change-points: 14.6, 48.1 and 53.6 Mb) (Figs 2A and S3 and S4 Table). The highest recombination rate at 2.2 Mb (*ρ*/Kb = 0.8) occurred at the distal end of the chromosomes, farthest from the PAR boundary. In the mid-PAR, there was a recombination valley containing the minimum rate in the PAR at 22.3 Mb (*ρ*/Kb = 0.02). Examining the 5 Mb region immediately adjacent to the PAR boundary showed that the recombination rate was significantly higher than the autosomal average (mean (SD): *ρ*/Kb = 0.19 (0.05)) (Mann-Whitney U: U = 1463, *p* = 2.175e-06). In contrast, within the SLR the recombination rate dropped abruptly to an average of 0.1 *ρ*/Kb (SD = 0.089), consistent with a lack of recombination in females.

The recombination rate influenced LD patterns across the Z chromosome (Fig 2C). Regions with higher recombination rates had reduced levels of LD (Generalized Least Squares of LD~recombination rate: *t* = -8.1, *p* < 0.001), with LD declining rapidly with recombination rate (Fig 2E). The mean pairwise LD for the whole PAR was similar to that of autosomes (mean (SD) PAR = 0.132 (0.017), autosomes = 0.134 (0.017)) and reached a ~50 Kb pairwise SNP distance in the PAR and ~65 Kb in the autosomes. There was, however, a difference in LD decay between sections of the PAR according to their distance to the PAR boundary, consistent with changes in recombination rate (Fig 2F). Across the 200 Kb region spanning the PAR boundary, there was little LD between the PAR and SLR (Fig 2C), indicating that even PAR loci in close physical proximity to the PAR boundary (within ~50Kb) effectively segregate independently from the SLR.

The higher recombination rate in the regions closest to and farthest from the PAR boundary may be caused by cross-over pairing between the Z and W being forced into a smaller region in females than in males due to W chromosome degeneration [37]. To investigate whether females are driving the overall recombination rate in the PAR we used genetic map data from [10] (Fig 2B). Female recombination rate for the PAR (mean (SD) = 1.70 (1.8) cM/Mb) was indeed significantly higher than the male rate (mean (SD) = 0.85 (0.93) cM/Mb, Mann-Whitney U: U = 1635, *p* = 0.025). Within the 5 Mb region closest to the PAR boundary, the female recombination reached 3.41 cM/Mb, while the male recombination rate was only 0.41 cM/Mb. Furthermore, *ρ* was significantly correlated with sex-averaged genetic map recombination rate (cM/Mb) across the PAR (R = 0.32, *p* = 0.02), and this was driven by female, not male recombination rate (Correlation with female map cM/Mb: R = 0.35, *p* = 0.013, Correlation with male map cM/Mb: R = 0.11, *p* = 0.45, Fig 2D). Together these results imply that historical recombination rates of the ostrich PAR are shaped by the recombination rate of heterogametic females.

### Patterns of genetic diversity and female-male divergence in allele frequency across the sex chromosomes

**Fig 3:**
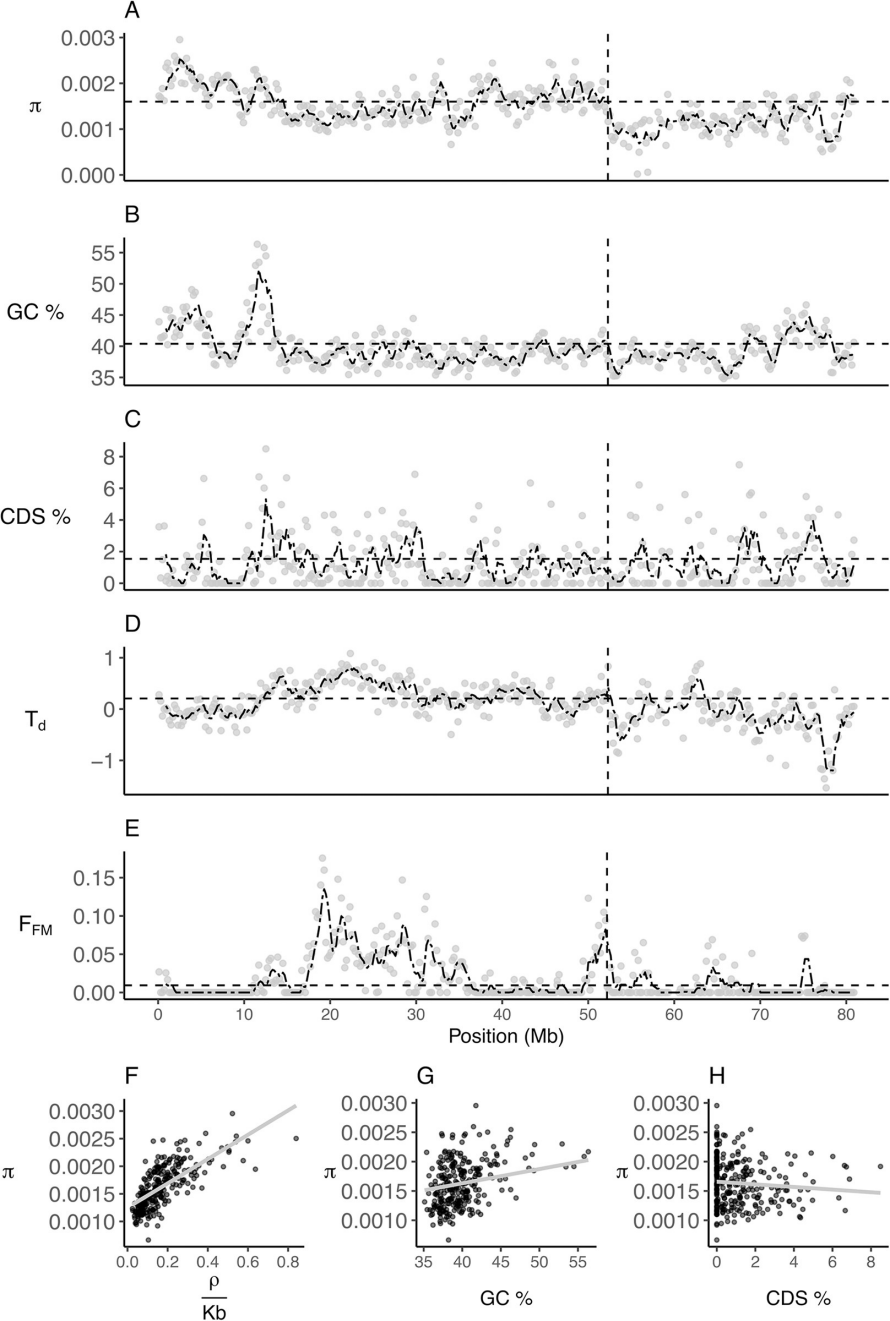
Population genomic features were highly variable across the ostrich Z chromosome. (A) Pairwise nucleotide diversity (π), (B) GC content (%), (C) Coding DNA Sequence (CDS) density (%), (D) Tajima’s D (T_d_) and (E) Female-male *F*_ST_ (*F*_FM_), calculated for 200-Kb non-overlapping windows (Gray points). Pairwise nucleotide diversity on the PAR plotted as a function of (F) population scaled recombination rate (*p* < 2.2e-16), (G) GC content (*p* = 0.0018) and (H) CDS density (*p =* 0.041). *p*-values are for GLS regression coefficients of each explanatory variable taken from the full model reported in the text. In (A) to (E), the black dash-dotted line is the rolling average calculated over a span of ~1 Mb, the horizontal dashed line indicates the autosomal average and the vertical dashed line indicates the PAR-SLR boundary at ~52.2 Mb.

Genetic diversity across the Z chromosome was variable with a clear break at the boundary between the PAR and the SLR (Fig 3A). The average level of genetic diversity in the PAR was similar to autosomal levels (mean (SD) π: PAR = 0.0016 (0.0004), autosomes = 0.0016 (0.0004), Mann-Whitney U: PAR vs autosomes, U = 97478, *p* = 0.1). In the SLR, the genetic diversity dropped abruptly (mean (SD) π = 0.001 (0.0002)), as expected with complete cessation of recombination (Fig 3A) and was significantly lower than within the PAR (Mann-Whitney U: PAR vs SLR, U = 5615, *p* = 1.349e-09). The heterogenous pattern of genetic diversity across the PAR was correlated with recombination rate (Fig 3F. GLS of genetic variation ~ *ρ*/Kb: t = 10.22, *p* < 0.001) and GC content (Fig 3B and 3G. GLS: t = 2.51, *p* = 0.013). This positive correlation with GC content is potentially due to GC-biased gene conversion during recombination events [20]. Conversely, genetic diversity was negatively related to gene density, although this relationship was weak (Fig 3C and 3H. GLS: t = -1.89, *p* = 0.06). These results are consistent with the idea that linked selection in regions with high gene density and low recombination rates leads to reduced genetic diversity [38,39].

We examined if there were regions of the PAR with allele frequency differences between females and males using two different measures, Tajima’s D and *F*_ST_ between females and males (*F*_FM_). Partial linkage with the SLR causes neutral variants to have deeper coalescent times, resulting in a signature of elevated diversity resembling balancing selection, as indicated by higher values of Tajima’s D [15]. We found Tajima’s D was similar in the PAR and autosomes (Mann-Whitney U: U = 84963, *p* = 0.1), and not significantly higher at the PAR boundary, as expected if allele frequencies are not influenced by the SLR. Instead, Tajima’s D was highest in the mid-PAR with the maximum value of 1.1 at 22.3 Mb (Fig 3D). Similarly, values of *F*_FM_ were highest mid-way through the PAR (Fig 3E). The average *F*_FM_ across the PAR was also significantly higher than both autosomes and the SLR (Mann-Whitney U: autosome–PAR, U = 65673, *p* <0.001; PAR–SLR, U = 23558, *p* = 1.646e-08). The mid-PAR had the lowest recombination rate and genetic diversity, which might explain the elevated values of divergence in female-male allele frequency.

### Are patterns across the PAR consistent with neutral genetic theory?

**Fig 4:**
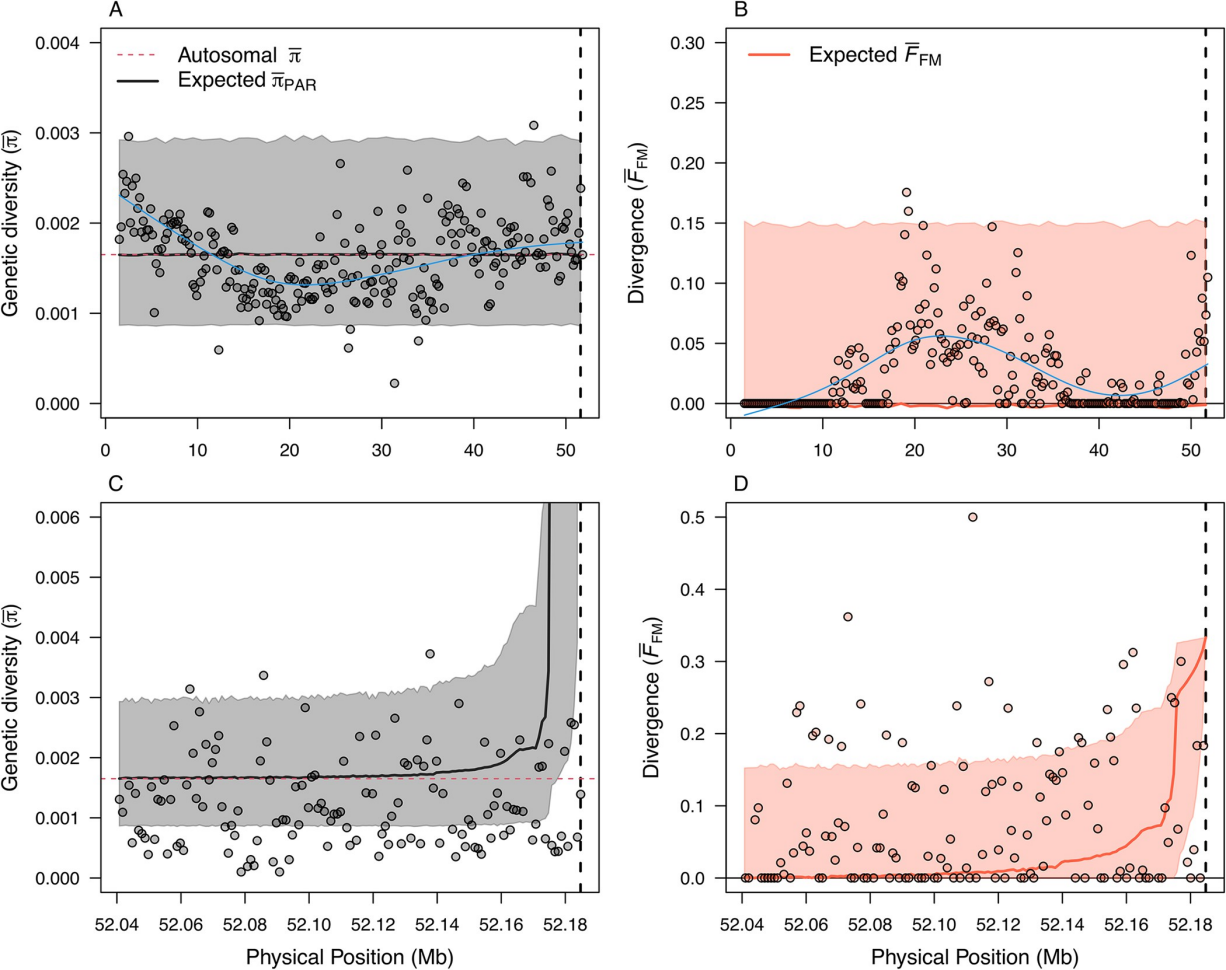
Predicting genetic diversity and female-male divergence across the ostrich PAR using neutral genetic theory. **(**A) Predicted average neutral genetic diversity, $\bar{\pi }$, and (B) female-male divergence, *F*_FM_, across the full PAR (Physical position 0–52.2 Mb). Solid lines indicate the mean value of 1,000 replicate coalescent simulations (Methods section “Coalescent simulations"), with shading indicating 95% confidence intervals (i.e., 95% of calculated values from the simulations fell within this interval). Points indicate empirical estimates calculated for 200 Kb windows. Panels (C) and (D) are high resolution illustrations (1 Kb windows) of the ~140 kb region immediately adjacent to the PAR boundary.

To test if the observed patterns of genetic diversity and female-male divergence across the PAR are consistent with neutral genetic processes, we used coalescent simulations based on [15]. Our results were broadly consistent with neutral genetic theoretical predictions of the expected average pairwise nucleotide diversity ($\bar{\pi }$), with empirical estimates falling within the 95% confidence intervals from coalescent simulations (Fig 4. See Materials & Methods: Coalescent simulations). Genetic diversity did, however, exceed the 95% confidence intervals in two 200 Kb windows, one at the start of the chromosome and one at the PAR boundary (Fig 4A). Focusing on the small region around the PAR boundary (~10–20 Kb), our simulations predicted a nonlinear increase in $\bar{\pi }$ (Fig 4C, solid line). This was not seen in our empirical estimates, where $\bar{\pi }$ was lower than expected adjacent to the PAR boundary (Fig 4C), and higher farther away.

Examining patterns of female-male divergence showed a region in the middle of the PAR (~15 to ~25 Mb) with elevated *F*_FM_ values that fell outside the 95% confidence interval of the simulations (Fig 4B). There was also a suggestive spike in *F*_FM_ very close to the PAR boundary (Fig 4D). Our simulations also predicted a sharp increase in *F*_FM_ in the small region adjacent to the PAR boundary, similar to patterns of genetic diversity. However, the windows with elevated *F*_FM_ values, that exceeded theoretical predictions, were too far away from the SLR to be sex-linked (between positions 20-30Mb on the Z). Overall, it appears that the high recombination rates in females, especially in the region adjacent to the PAR boundary (see Fig 2B), rapidly break down genetic associations between the SLR and PAR loci, resulting in empirical patterns of genetic diversity that are consistent with predictions from neutral theory.

## Discussion

The maintenance of recombination in ancient sex chromosomes, as observed in ratites, is one of the long-standing conundrums of sex chromosome evolution. In this study, we used population genomic data from ostriches to investigate how patterns of recombination and genetic variation across the sex chromosomes change in relation to the SLR. We found that the genetic linkage between the SLR and the PAR was restricted to an extremely small region (~50Kb), which means that most of the PAR segregates independently from the SLR.

The PAR sequence that is tightly linked to the SLR is estimated to occur within a cumulative *ρ* = 1 from the PAR boundary in heterogametic females [15]. For the 200 Kb window adjacent to the PAR the sex-averaged population scaled recombination rate was 127.1. This means that the region where *ρ* = 1 is predicted to be only ~1575 base pairs long. The length of this region is comparable to that found in *Silene latifolia* where only ~500 base pairs of the PAR were estimated to be in strong LD with the SLR [40]. Additionally, in our study, genetic variation was not above the autosomal mean, as expected if the PAR loci were in linkage with the SLR. While these results suggest that LD decays extremely fast as you move away from the PAR boundary, it is possible that the difference between the theoretical predictions from our simulations and our empirical estimates is due to low SNP densities near the PAR boundary, or possibly because there is an assembly gap of ~7.6 Kb between the PAR and the SLR (S1 Fig).

The recombination rates of the ostrich PAR and autosomes were comparable to studies on two other ratites. In the greater rhea (*Rhea americana*), the PAR is 52.5 Mb and the average recombination rate was similar to that of autosomal pairs 5 and 6 [35]. In the emu (*Dromaius novaehollandiae*), a comparison of recombination rates between 14 PAR and 8 autosomal loci concluded that recombination rates are slightly higher, and LD slightly lower, in the PAR than the autosomes [41]. In this study, patterns of recombination were highly variable along the PAR. In addition to the effects of the PAR boundary, recombination variation was consistent with the possible localization of cross-overs towards the chromosome ends [42]. Interestingly, we found that variation in historical rates of recombination of the PAR was largely explained by patterns of recombination in females and not males (Fig 2D). A higher recombination rate in the heterogametic sex has been hypothesized to protect the PAR from degeneration, enabling the maintenance of long PARs over prolonged evolutionary time periods [3]. In the case of ostrich, it seems that the pairing of the W and Z also causes higher recombination rate in this region.

Another feature of the PAR was a recombination valley in the middle section which coincided with a reduction in genetic diversity, a positive Tajima’s D and elevated levels of female-male allelic divergence (Fig 3E). While the elevated female-male divergence might hint at the action of sexually antagonistic selection, coalescent simulations showed the observed patterns are still consistent with neutral predictions. We propose that this saddle-shaped pattern of recombination is consistent with the combined effects of: (i) enforced pairing between the Z and W chromosomes both at the distal end and close to the PAR boundary, and (ii) cross-over interference limiting recombination rates in the mid-PAR [37].

In systems with smaller PARs, such as humans [25] and collared flycatchers [5], there is little empirical support for the role of sexual antagonism in shaping recombination patterns of sex chromosomes (although confirming the role of sexually antagonistic selection is extremely challenging). Small PARs, however, have extreme recombination dynamics that make it difficult to confirm or deny the importance of sexually antagonistic polymorphisms in the degeneration of the sex-limited chromosome. Species with long PARs offer opportunities to study the influence of the SLR on sex chromosome evolution without such complications. In the large PAR of the recently evolved sex chromosomes of *Silene latifolia*, eight genes with positive Tajima’s D were detected [40]. Two of these genes, that were closest to the SLR, had different female-male allele frequencies, but the other six genes that were loosely linked to the SLR did not show any sex differences in allele frequencies. A simulation study on these six genes concluded that the positive Tajima’s D could not be explained solely by demography, and that sexually antagonistic selection might be responsible for creating the observed patterns under a scenario where they were historically closely linked to the SLR [43].

The recombination of ancient ratite sex chromosomes may be maintained by several processes, including sex-biased gene expression [3], a slower rate of molecular evolution [44], and a high recombination rate at the PAR boundary reducing LD between the SLR and PAR loci [3]. In ostriches, it is unlikely that the sex chromosomes are maintained due to sex-biased gene expression, as the genes on the PAR in adults are equally expressed in both sexes [45]. Ratites, including the ostrich, do however have a slower rate of molecular evolution [46] making it possible that this has decreased the accumulation of genetic mutations that degenerate the W chromosome. Our study also highlights that a high recombination rate at the PAR boundary might prevent the accumulation of sexually antagonistic mutations. The theory for the evolution of recombination suppression of sex chromosomes due to sexual antagonism relies on sexually antagonistic alleles building genetic associations with the SLR. A key insight from our results is that an elevated recombination rate at the PAR boundary can greatly restrict the size of the region where sexually antagonistic alleles can become genetically associated with the SLR, providing an explanation for the evolutionary maintenance of ancient recombining sex chromosomes.

## Materials and methods

### Ethics statement

All procedures were approved by the Departmental Ethics Committee for Research on Animals (DECRA) of the Western Cape Department of Agriculture, reference no. AP/BR/O/SC14.

### Study population, sampling, and sequencing

Blood samples of *Struthio camelus* were obtained from Western Cape Department of Agriculture’s ostrich research facility in Oudtshoorn, South Africa. Since 1995, individuals have been bred in pairs at the research facility to create pedigrees. At the time of sampling, the pedigrees contained 1531 males and 2067 females. We selected 5 males and 5 females for sequencing using the program PedMine [47]. PedMine identifies individuals with most distant links within pedigrees allowing the maximum amount of genetic diversity in populations to be sampled. Samples were sequenced at Science for Life Laboratory, the National Genomics Infrastructure, using paired end with 126 base pairs on Illumina HiSeq 2500, following manufacturer’s protocol.

### Mapping, variant calling and filtering

We implemented a snakemake [48] workflow for mapping and variant calling. Briefly, reads were trimmed with cutadapt version 2.10 [49] and then mapped to the optical map improved reference genome (Struthio_camelus.20130116.OM.fa) with bwa version 0.7.17.r1188 [50]. Mean coverage per sample is presented in S1 Table. Duplicates were marked with Picard MarkDuplicate [51]. The ostrich Z chromosome in the assembly version used in this study consists of 12 scaffolds (S2 Table). By measuring the average male and female coverage, the coordinate of the PAR boundary was determined to be in superscaffold36 between 3516672 and 3524264 with gap size of 7592 nucleotides (S1 Fig). We checked for the existence of gametologous genes on the 8 SLR scaffolds by identifying annotated genes with a copy on a putatively W-linked scaffold. We measured the male to female coverage ratio for the putative W-linked scaffold and if the ratio was close to zero, we determined the scaffold to be W-specific and containing gametologous gene (S3 Table). Heterozygous SNPs in females overlapping with these genes were removed from further analyses since they reflect divergence between Z and W since recombination cessation.

Variant calling was performed with GATK version 4.1.4.1 following best practice procedures developed at the Broad Institute [52]. The GATK HaplotypeCaller was run individually on each sample to generate GVCF output. GVCF files for all samples were imported to a GenomicsDB datastore, followed by genotyping with GATK GenotypeGVCFs to produce a final raw variant call set. Several filtering steps were performed on the raw call set to obtain the final call set of high quality. Biallelic SNPs were selected with GAKT SelectVariants and filtered with GATK VariantFilteration using best practice options QUAL < 30, QualByDepth (QD) < 2.0, RMSMappingQuality (MQ) < 40.0, MappingQualityRankSumTest (MQRankSum) < -12.5, FisherStrand (FS) > 60.0, ReadPosRankSumTest < -8.0 and StrandOddsRatio (SOR) > 3.0. We removed variants overlapping with repeats annotated by the *aves* repeat library using BEDTools intersect [53]. We filtered SNPs with more than twice the average coverage (>70 reads) and less than 5 reads per site. SNPs in the SLR in females are expected to occur only as haploid. However, heterozygous SNPs in the SLR in females can occur either due to genotyping error or due to the divergence of the Z and W sequences in the gametologous region. We therefore filtered the heterozygous SNPs in females in the SLR. This left us with 5,776,166 SNPs for autosomes, 268,006 SNPs for the PAR and 89,540 SNPs for the SLR. Distribution of alternative allele frequency and per site depth is shown in S2 Fig. To filter the background non-variant sites, we calculated coverage per site using samtools version 1.14 [54]. We used the hard-masked reference genome for repeats and filtered sites that had a minimum of 5 reads or a maximum of 70 reads. Coverage filter removed 78,235,359 sites from the whole genome. VCF files are publicly available in Dryad database [55].

### Measures of population scaled recombination and linkage disequilibrium

Pedigree-based recombination rate provides us with an estimate of the recombination rate for one generation, but population scaled recombination rate (*ρ* = 4*N*_**e**_*r*) gives us an estimate of recombination in the history of sample. Population scaled recombination rate was estimated for each Z scaffold separately in windows of 1000 SNPs with an overlap of 200 SNPs. The *interval* program in LDhat 2.2 [36] was used, and three independent Markov-Chain Monte Carlo (MCMC) chains were run with a block penalty of 5 and 25 million iterations. We sampled the chain every 5000 iterations and discarded the first fifth (5,000,000 iterations) as burn-in. To determine where the trend in population scaled recombination rates changes, we performed change-point analysis using the *segmented* package in R [56]. The change-point analysis with sex-averaged population scaled recombination rate returned 3 significant change-points along the graph, at 14.6, 48.1 and 53.6 Mb (S3 Fig). We have used these change points to define four regions: the SLR where recombination rate drops to an average of 0.1; the 5 Mb segment closest to the PAR boundary where recombination is higher than autosomal average; the mid-PAR containing the minimum recombination rate at 22.3 Mb, and the region most distant from the PAR boundary where recombination frequency reaches its maximum value well above the autosomal average at 2.2 Mb (S4 Table). Pairwise linkage disequilibrium (LD) was measured as the square of the correlation coefficient between the allelic states (*r*^*2*^) for all pairs of SNPs within 200 Kb window with 50 Kb overlap after filtering for Hardy-Weinberg equilibrium (HWE) in vcftools [57] using PopLDdecay [58].

### Measures of genetic variation and female-male allelic differentiation

Pairwise nucleotide diversity (*π*), the number of segregating sites (θ) and the relationship between the two, measured as Tajima’s D statistic [59] for neutrally evolving sequences were calculated across chromosome Z in 200 and 1000 Kb non-overlapping windows using vcftools version 1.16 [57] and custom Python scripts located under https://github.com/Homap/ostrich_PAR_analysis/tree/main/code/analysis/diversity. All population genetics measures were calculated for the SLR considering its haploid state in females. We investigated the relationship between genetic variation in the PAR with recombination rate, GC content and gene density using Generalized Least Squares (GLS) regression with autocorrelation structure (corAR1), and maximum likelihood estimation using the nlme R package [60]. We measured genetic differentiation between females and males using *F*_ST_ measure of population differentiation [61] in vcftools version 1.16 [57].

### Coalescent simulations

We modeled the expected neutral genetic diversity (π) and between-sex divergence (female vs. male *F*_*ST*_) for the ostrich PAR following the approach of [15] and [16]. All coalescent simulations were performed using the computationally efficient simulator msprime [62]. We leveraged the fact that the coalescent for recombining sex chromosomes is mathematically equivalent to the structured coalescent for two demes (representing X and Y or Z and W chromosomes) where recombination causes migration of genes between demes. When applied to the ostrich Z-W sex chromosomes system, the effective population sizes for the two demes corresponding to Z and W chromosomes are 3*N*_*e*_/4 and *N*_*e*_/4, respectively, where *N*_*e*_ is the effective population size for an autosomal gene. The forward recombination rate in females (movement of a gene from a Z to a W chromosome) is denoted *r*_*f*_, while the backward rate (moving from a W to a Z chromosome) is equal to *r*_*f*_/3 [15]. Recombination in males only shuffles genes between Z chromosomes (i.e., within deme), and therefore does not influence coalescence times. The population-scaled recombination rate was calculated as 4*N*_*e*_*r*_*f*_.

The key predictions from the models are the average coalescence times for genes sampled on two different Z chromosomes (${\bar{T}}_{ZZ}$), two W chromosomes (${\bar{T}}_{WW}$), and on a Z and W chromosome (${\bar{T}}_{ZW}$), from which we can calculate the average coalescence time for alleles sampled within a deme (${\bar{T}}_{w}=({\bar{T}}_{ZZ}+{\bar{T}}_{WW})/2$) and average total coalescence time (${\bar{T}}_{t}=\frac{\left({\bar{T}}_{ZZ}+{\bar{T}}_{WW}\right)}{4}+\frac{{\bar{T}}_{ZW}}{2}$). Average coalescent times for genes sampled in males and females can be calculated in similar fashion. The average genetic diversity at a given site located within the PAR will be proportional to ${\bar{T}}_{t}$ such that, in the limit of high recombination (*r*_*f*_ ➔ ½), ${\bar{T}}_{t}$ for genes located in the PAR will converge on that of autosomal genes [15]. The expected genetic diversity for a site at a given location within the PAR (i.e., with *N*_*e*_ and *r*_*f*_ values determined by the physical position of a given gene) will be, approximately, ${\bar{\pi }}_{PAR}={\bar{\pi }}_{Auto}{\bar{T}}_{t}$. The average between-sex divergence will be approximately equal to $F_{ST}^{fm}\approx 1-\frac{{\bar{T}}_{w}^{fm}}{{\bar{T}}_{t}^{fm}}$, where ${\bar{T}}_{w}^{fm}=({\bar{T}}_{ff}+{\bar{T}}_{mm})/2$ is the expected within-chromosome coalescence time for a pair of genes sampled in males and a pair sampled in females, and ${\bar{T}}_{t}^{fm}=({\bar{T}}_{ff}+{\bar{T}}_{mm}+2{\bar{T}}_{fm})/4$ is the expected total coalescence time for a pair of genes where one is sampled from a female and the other is sampled from a male [16].

To generate the theoretical expectations presented in Fig 4, we performed 1000 replicate coalescent simulations for each of 200 evenly located sites on the Z chromosome, starting at the PAR boundary and extending toward the distal end of the chromosome arm (encompassing all 52 MB of the PAR for analyses of the full PAR, and for only the 144 Kb immediately adjacent to the PAR boundary) using the same number of sampled chromosomes from females and males as were present in the empirical data set. For each site we used the population scaled recombination rate (*ρ*) as described above. Using *ρ* and pedigree-based recombination frequency (*r)*, we obtained estimates of *N*_*e*_ across the PAR following $N_{e}=\frac{\rho }{4r}$. We estimated recombination frequency from the genetic map obtained in [10] using the Kosambi map function $r=\frac{1}{2}(\frac{e^{4x}-1}{e^{4x}+1})$, where *r* is the recombination fraction and x is the average number of crossovers. The unit of map distance measured this way is Morgan (M), one Morgan is defined as the length of a chromosome segment bracketed by two loci that produces, on average, one crossover per meiosis. For each site, we calculated ${\bar{\pi }}_{PAR}$ and *F*_*FM*_ from replicate simulations; confidence intervals were calculated as the 2.5 and 97.5 percentiles for each metric across replicate simulations. All computer code needed to reproduce the simulations, population genomics analyses and data processing are available in the GitHub repository (https://github.com/Homap/ostrich_PAR_analysis).

### Dryad DOI

10.5061/dryad.pnvx0k6sx.

## Supporting information

S1 Table Median read coverage per sample. (XLSX)

S2 Table List of Z-linked scaffolds in ostrich assembly. (XLSX)

S3 Table List of putative gametologous genes and their coordinates along the SLR Z-scaffolds and W-linked scaffolds. (XLSX)

S4 Table Change-point analysis for population scaled recombination rate along the Z chromosome. (XLSX)

S1 Fig Coverage in males (blue) and females (red) in the PAR-SLR boundary. The boundary is located on superscaffold36. Dashed lines indicate the boundary coordinates used for this study (superscaffold36: 3,516,672–3,524,264).
(TIF)

S2 Fig Distribution of alternative allele frequency (top row) and read depth per site (bottom row) for the PAR, autosomes and SLR. (TIF)

S3 Fig Change-point analysis across the Z chromosome for population scaled recombination rate. Three significant change points were identified at 14.64, 48.1 and 53.6 Mb (red circles).
(TIF)
