# Supplementary figures and images for: The evolutionary maintenance of ancient recombining sex chromosomes in the ostrich
Source: PLoS Genet. 2023 Jun 30;19(6):e1010801. doi: 10.1371/journal.pgen.1010801 (PMC10343094; doi:10.1371/journal.pgen.1010801)

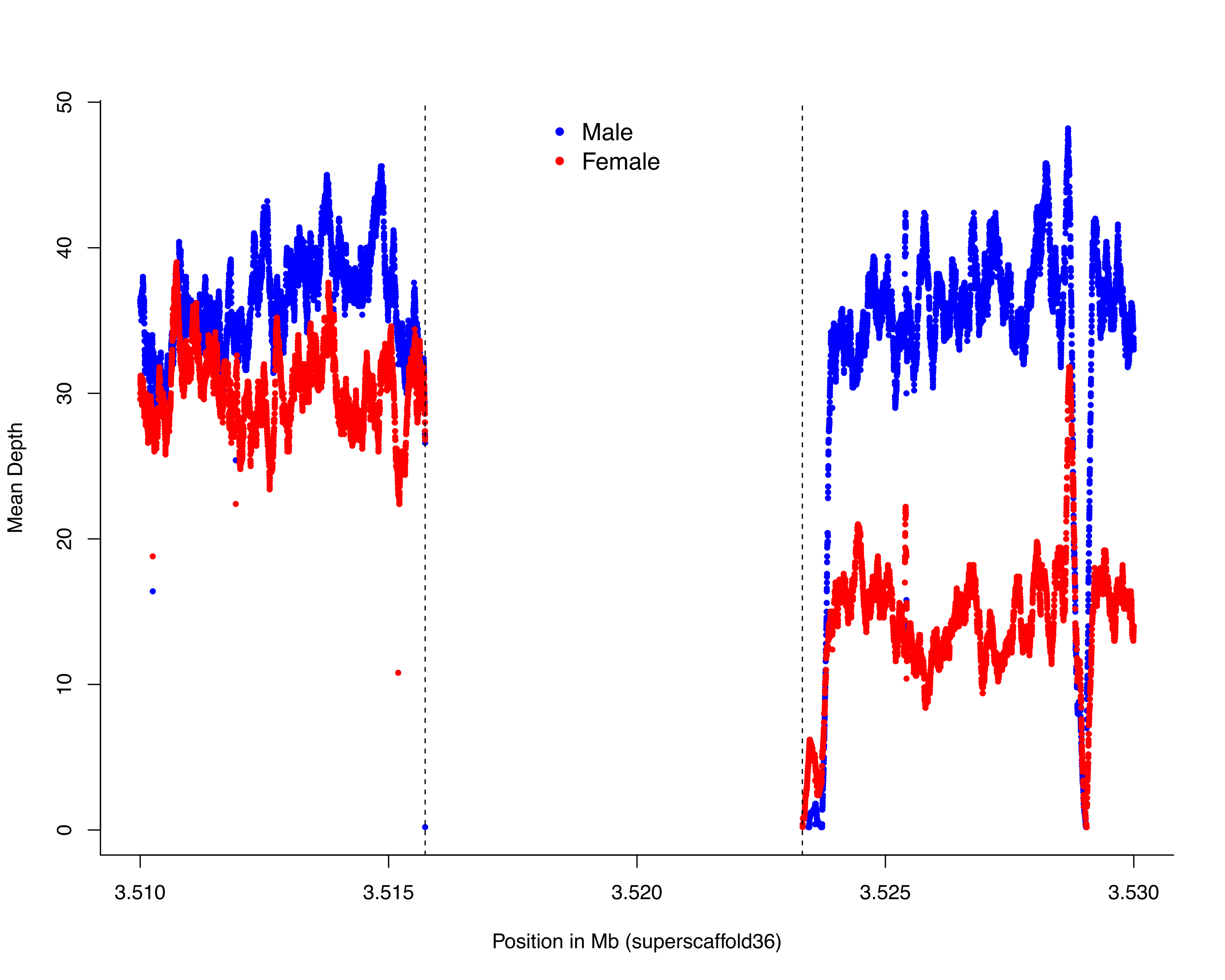

Supplement: S1 Fig — The boundary is located on superscaffold36. Dashed lines indicate the boundary coordinates used for this study (superscaffold36: 3,516,672–3,524,264). (TIF) [file pgen.1010801.s005.tif]

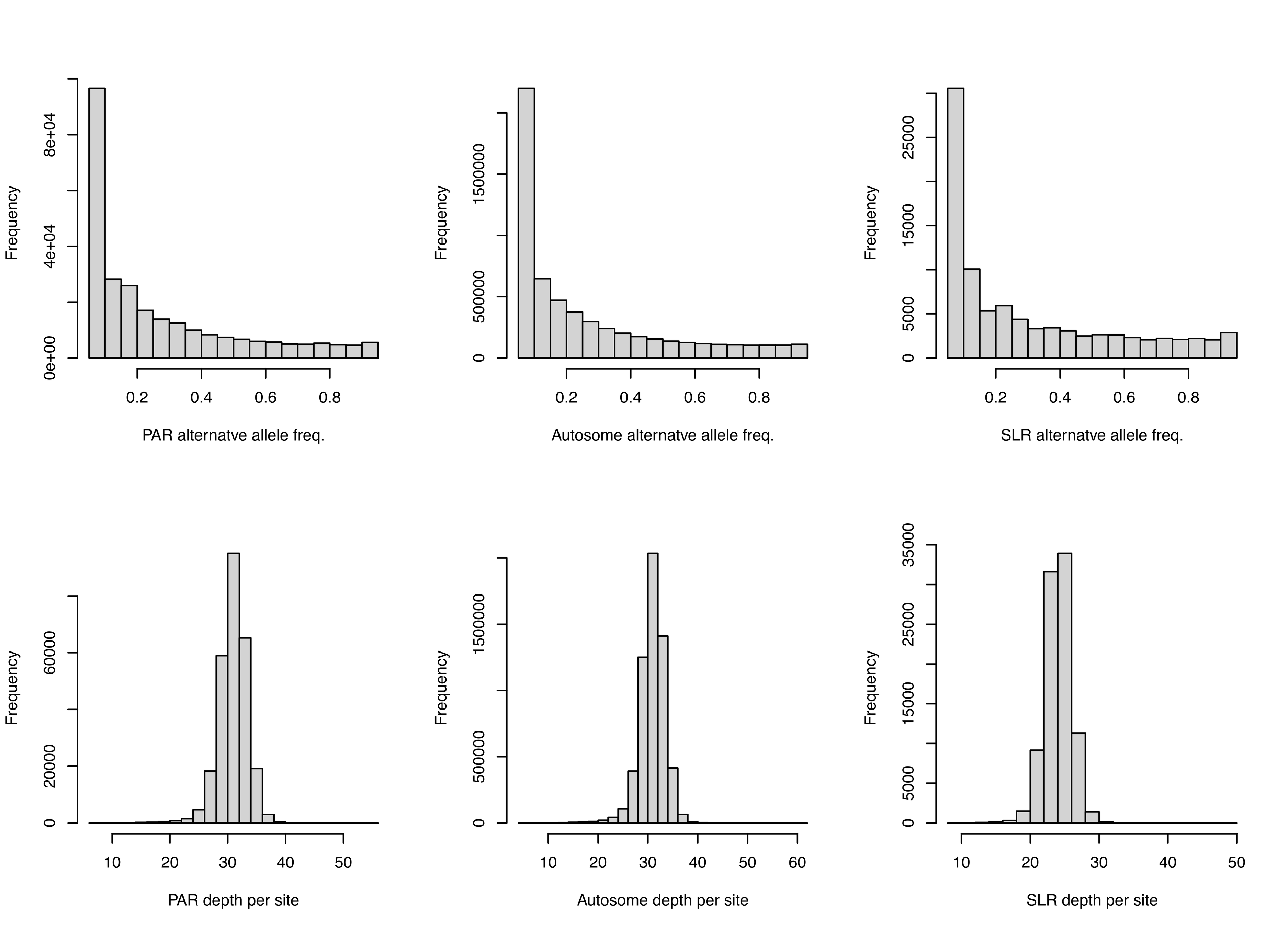

Supplement: S2 Fig — (TIF) [file pgen.1010801.s006.tif]

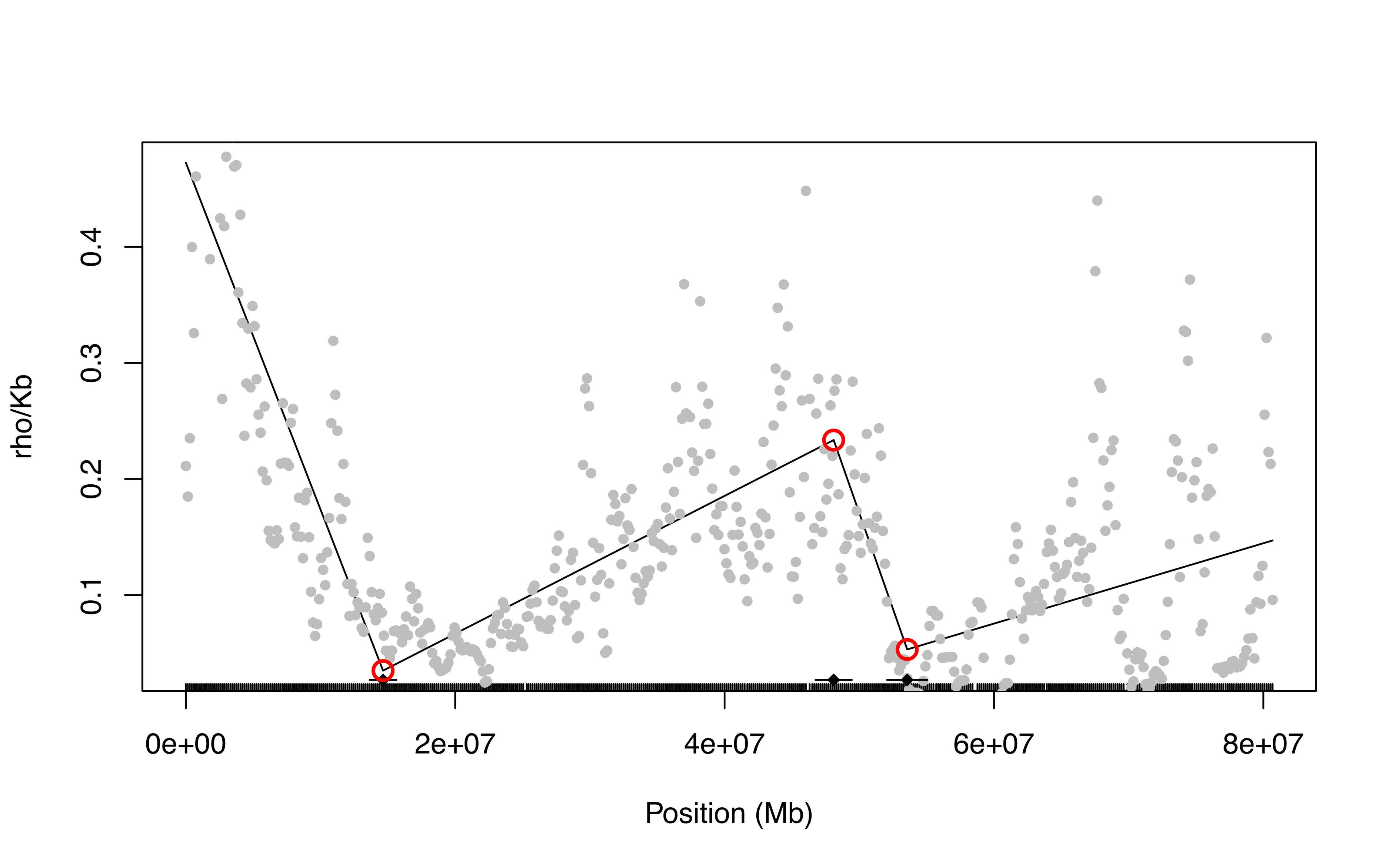

Supplement: S3 Fig — Three significant change points were identified at 14.64, 48.1 and 53.6 Mb (red circles). (TIF) [file pgen.1010801.s007.tif]
